# The strategy of removing tracheal stents by using an interventional technique under fluoroscopy

**DOI:** 10.1186/s12890-022-02140-6

**Published:** 2022-09-14

**Authors:** Pengfei Xie, Shuai Wang, Xiaobing Li, Ying Liu, Yaozhen Ma, Mei-Pan Yin, Xinwei Han, Gang Wu

**Affiliations:** 1 Department of Interventional Radiology, The First Affiliated Hospital, Zhengzhou University, No.1, Jianshe Road, Zhengzhou, 450052 Henan China; 2 Department of Respiratory, The First Affiliated Hospital, Zhengzhou University, Zhengzhou, 450000 Henan China

**Keywords:** Trachea, Stenosis, Fistula, Stent, Stent removal, Interventional radiology

## Abstract

**Background:**

Tracheal stent implantation is widely used in clinic settings. Timely removal of tracheal stents could prevent or reduce related complications. This study was aimed at evaluating the feasibility and safety of removing tracheal stents by an interventional technique under fluoroscopy.

**Methods:**

Clinical data of patients with self-expanding uncovered tracheal stents removed by an interventional technique under fluoroscopy were analyzed retrospectively, including medical records, imaging findings, surgical records, and follow-up results. According to the type and time of stent placement and the proliferation of granulation tissue under bronchoscopy, different tracheal stent removal techniques were used to remove the tracheal stent under fluoroscopy, and the feasibility and safety of the interventions were analyzed.

**Results:**

In all, 148 tracheal stents were removed from 112 patients; 95.9% (142/148) of the stents were completely removed and 4.1% (6/148) had a small amount of metal residue, and foreign-body forceps were removed under fiber bronchoscopy guidance. In 78 (69.6%), 32 (21.6%), and 6 (5.4%) patients, the tracheal stent was removed by the internal stripping, direct removal, and stent-in-stent methods, respectively. The overall stent removal time ranged from 11 to 111 (28.9 ± 20.1) min. During stent removal, 16 (14.3%) and 13 (11.6%) patients developed mild and moderate complications, respectively. There were no serious complications such as massive hemorrhage, mediastinal fistula, or death.

**Conclusions:**

An interventional technique under fluoroscopy for stent removal is a feasible, safe, and effective method and could serve as a technique for tracheal stent removal in clinical settings.

## Introduction

Tracheal stent implantation is widely used in the treatment of benign and malignant tracheal stenosis or fistula [1–4]. Tubular airway metal stents are widely used in the clinical treatment of upper and middle tracheal stenosis or fistula, and have shown obvious therapeutic effects [4]. Long-term placement of tracheal stents can cause granulation tissue proliferation, resulting in tracheal restenosis, irritating cough, infection, bleeding, stent rupture or displacement, and other complications [5,6]. Timely removal of tracheal stents with due consideration of the clinical need for the stent could reduce or avoid related complications.

In recent years, rigid bronchoscopy under general anesthesia and fiberoptic bronchoscopy under local anesthesia have been used for the removal of metal tracheal stents. However, these techniques are associated with low success rates and many complications [7,8]. Small-sample cases of tracheal stent removal under fluoroscopy have also been reporte [4,9]. The aim of this study was to assess the feasibility and safety of tracheal stent removal by an interventional technique under fluoroscopy.

## Materials and methods

### Patients

Clinical data of patients who underwent removal of self-expanding braided tracheal stents under fluoroscopy in our center from November 2006 to September 2021 were retrospectively analyzed. All patients with benign airway diseases underwent covered stent implantation. In principle, patients with malignant airway diseases should receive uncovered stents. The data included medical records, imaging findings, surgical records, and follow-up results. The patients included had undergone braided tracheal stent removal via an interventional technique under fluoroscopy guidance. This study was approved by the ethics committee of the Zhengzhou University First Affiliated Hospital. Ethical approval code: 2022-KY-0024-002.

### Indications for stent removal

The indications for stent removal were as follows. **①** Achievement of treatment purpose: The stents were removed at an optional time to prevent the complications associated with long-term stent implantation. **②** Granulation tissue hyperplasia: After tracheal stent implantation, the patient developed excessive hyperplasia of tracheal granulation tissue, resulting in tracheal stenosis and dyspnea. **③** Stent displacement: The stent shifted after implantation, which resulted in ineffective resolution of tracheal stenosis or covering of the tracheal fistula. **④** Stent fracture: Stent rupture occurred after tracheal stent implantation.**⑤** Stent intolerance: Patients experienced severe foreign-body sensations after stent implantation and could not tolerate the stent, thereby necessitating its removal. **⑥** Inappropriate stent placement: The stent was immediately removed and placed in a suitable position in cases of inappropriately positioned stents.

### Stent removal technology

**Fig. 1:**
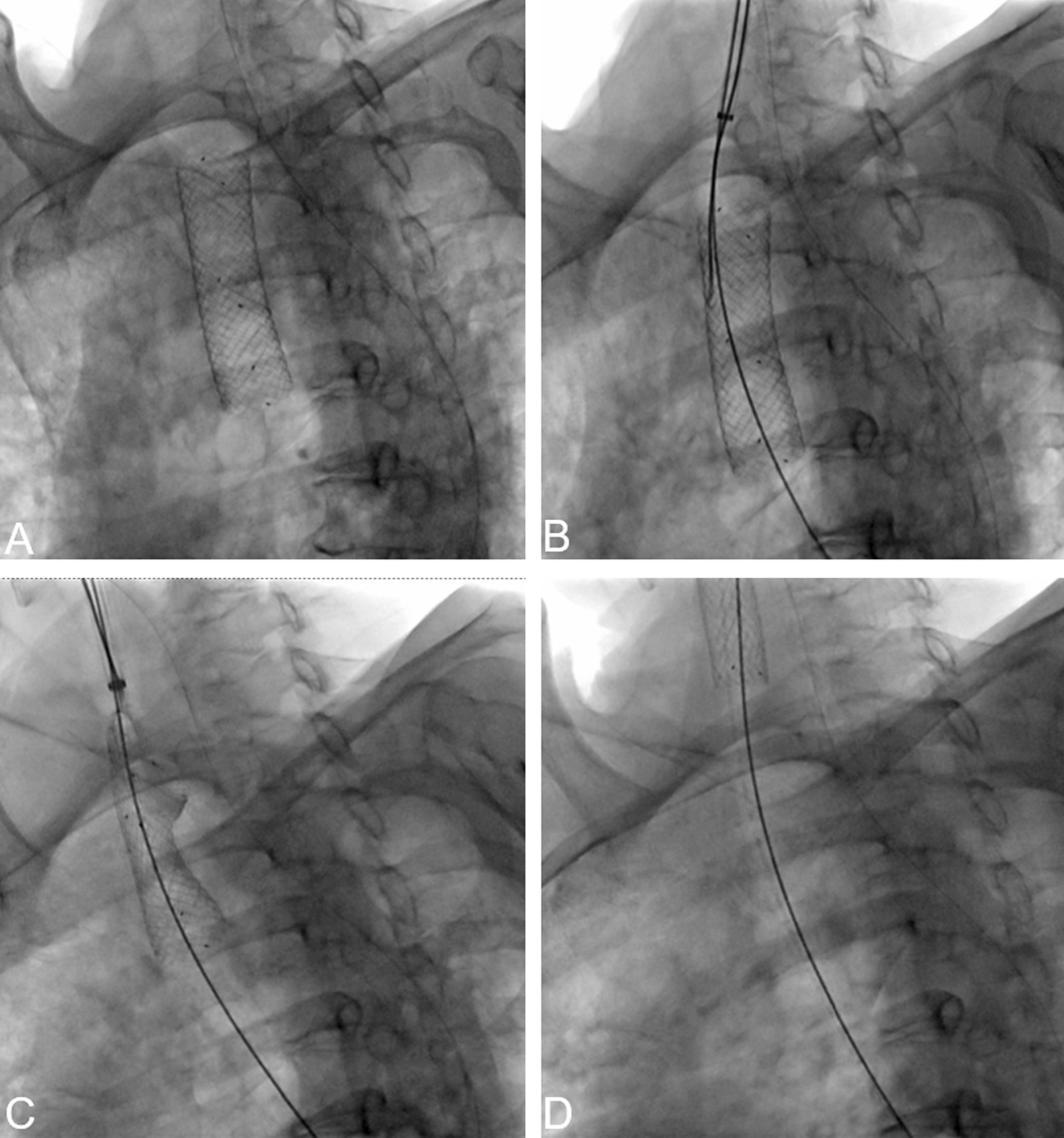
A 62-year-old female with tracheal stenosis caused by postoperative recurrence of esophageal cancer underwent tracheal covered stent implantation for more than 2 months. The patient subsequently experienced dyspnea for 7 days. CT showed that the tracheal stent had moved downward. DSA (**A**) showed that the tracheal stent had shifted downward. DSA (**B**) showed that stent-removal hook hooked the upper end of the tracheal stent. DSA (**C**–**D**) showed that the tracheal stent was then removed by direct extraction method, in which the stent-removal hook was attached to the upper end of the tracheal stent to collapse the proximal end. The patient’s dyspnea was relieved after the stent was removed

**Fig. 2:**
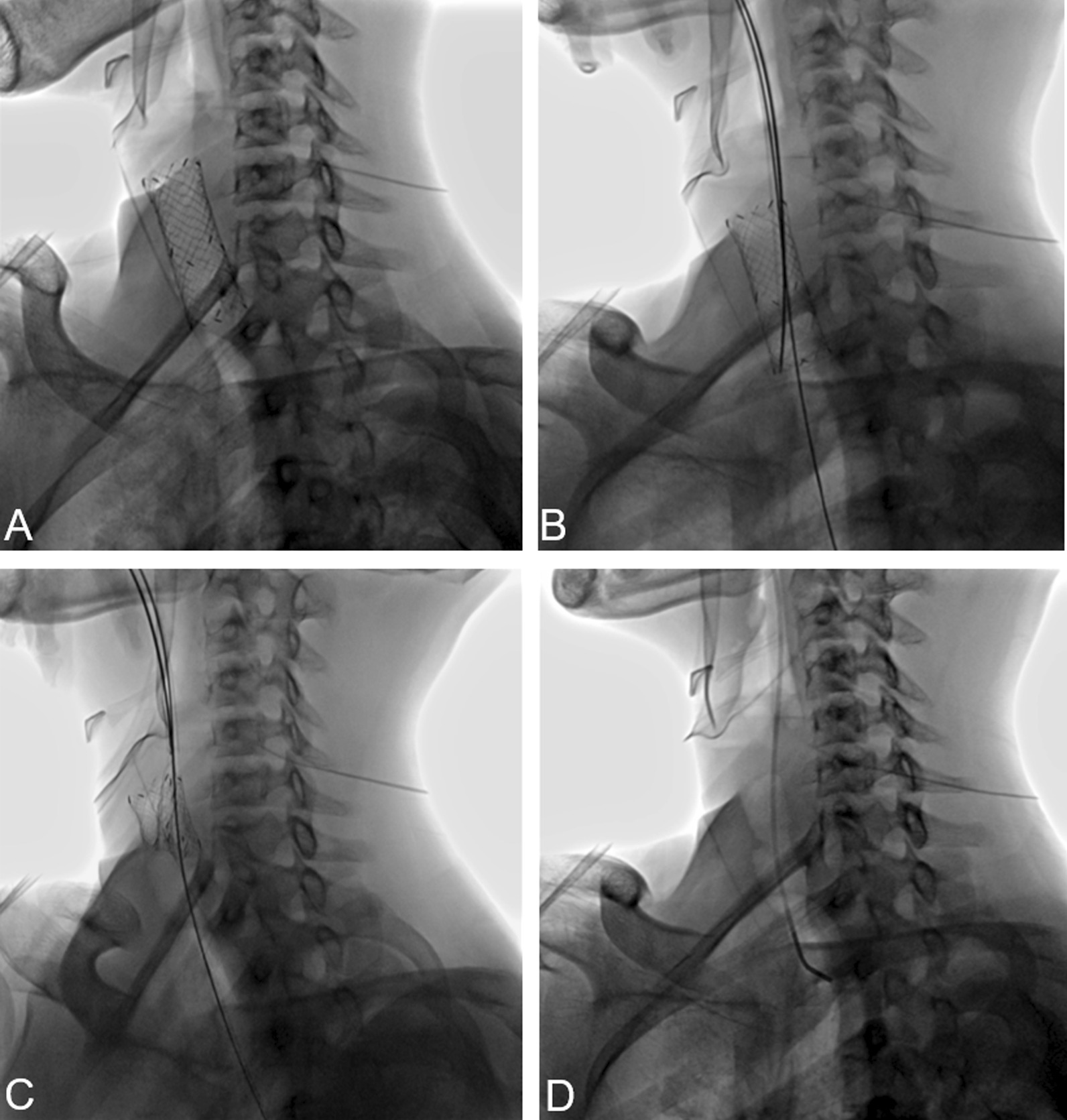
A 16-year-old male underwent tracheal covered stent implantation for more than 3 months due to benign tracheal stenosis. The patient subsequently experienced difficulty in breathing for more than 5 days. Bronchoscopy showed a large amount of granulation tissue at the upper and lower ends of the stent. DSA (**A**) showed that the tracheal stent was completely expanded in the main trachea. DSA (**B**) showed that stent-removal hook hooked the lower end of the tracheal stent. DSA (**C**–**D**) showed that the procedure involves hooking of the lower stent end with the stent taking out the hook, pulling of the stent taking out the hook outward, inward stent turning, and peeling off and pulling it out of the body. The patient’s dyspnea improved after the stent was removed

**Fig. 3:**
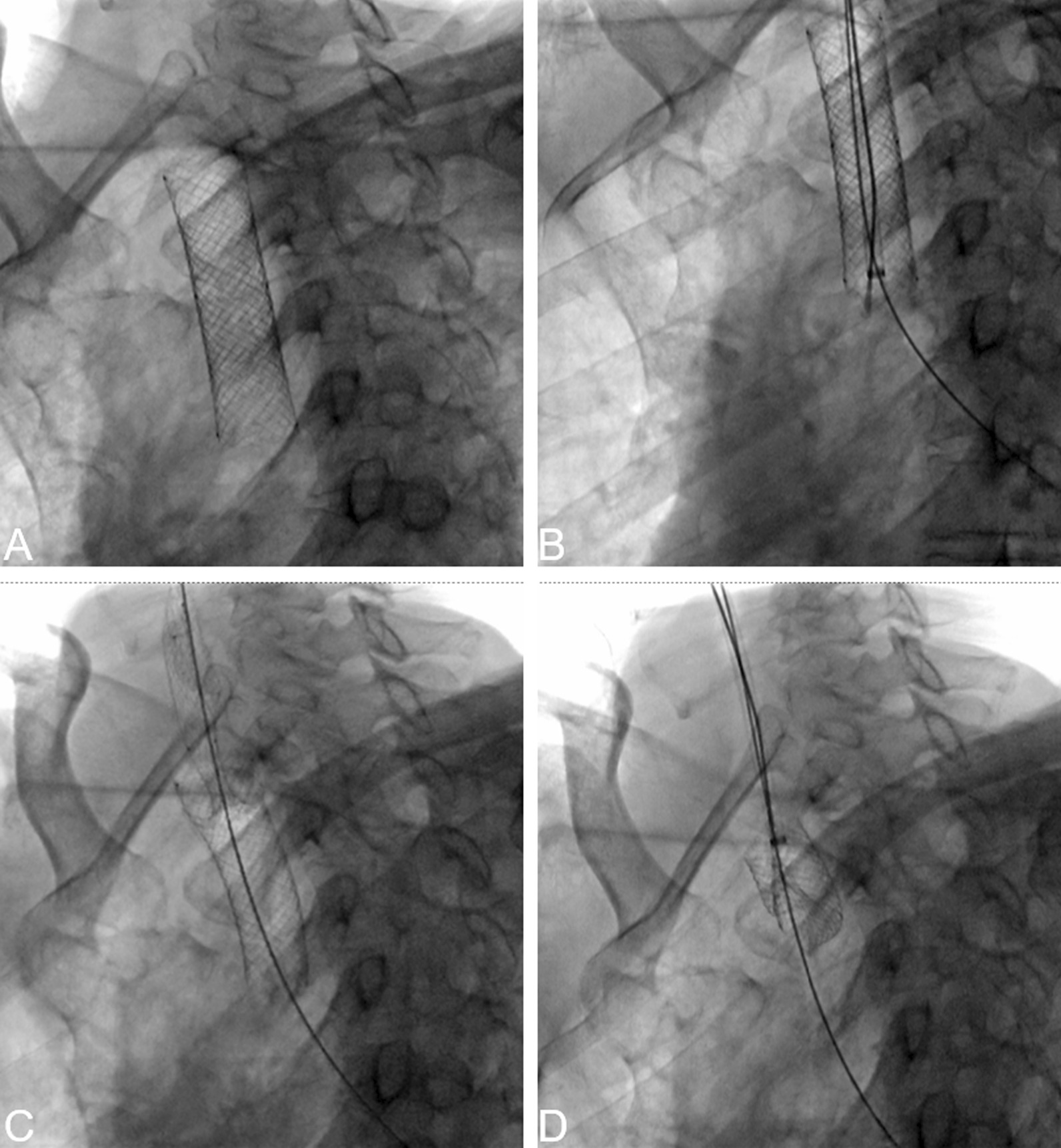
A 61-year-old man with tracheal stenosis due to postoperative recurrence of esophageal cancer underwent uncovered tracheal stent implantation for more than 3 months. Two months previously, the patient experienced dyspnea again, and bronchoscopy showed a large number of new organisms protruding into the metal mesh stent. A month ago, a covered stent was inserted into the original stent by the intervention technique. DSA (**A**) showed that the two tracheal stents were completely expanded in the main trachea. DSA (**B**) showed that stent-removal hook hooked the lower end of the inner tracheal stent. DSA (** C**) showed that inner tracheal stent was removed by internal stripping method. DSA (**D**) showed that external tracheal stent was removed by internal stripping method. The two stents were successfully removed. The patient's dyspnea improved significantly

**①** Direct extraction: This can be applied in cases with uncovered stents within 1 week of stent placement or those with covered stents without obvious granulation tissue proliferation. The estimated radiation exposure in the direct extraction method was 150–200 mGy. The procedure involves support removal, hooking of the upper support end, retraction and collapse of the proximal support end, and slow pull out of the body (Fig. 1). **②** Internal stripping: This is applicable for uncovered stents having a low or medium of granulation tissue proliferation at both ends of covered stents in patients without obvious dyspnea symptoms or low granulation tissue proliferation. The estimated radiation exposure in the internal stripping method was 180–250 mGy. The procedure involves hooking of the lower stent end with the stent taking out the hook, pulling of the stent taking out the hook outward, inward stent turning, and peeling off and pulling it out of the body (Fig. 2). **③** Stent-in-stent method^[[[10]]]^: This is applicable for covered stents with extensive granulation tissue hyperplasia at both ends of covered stents, and the stent-in-stent method is also applicable to uncovered stents with more than equal amount of granulation tissue hyperplasia. The estimated radiation exposure in the stent-in-stent method was 350–600 mGy. The procedure involves the insertion of a covered stent with the same diameter as the original stent or a stent having a diameter greater than 2 mm and length of at least 10 mm greater than those of the original stent, second stent removal by the direct removal method after 2–4 weeks, and first stent removal by internal stripping (Fig. 3).

### Stent removal procedures [4,9]

For patients with long-term stent implantation, bronchoscopy and chest CT were performed 1–3 days before the procedure to determine the proliferation of tracheal granulation tissue, whether the stent was broken, or whether there was tracheal stenosis or phlegm retention. Thirty minutes before stent removal, patients received an intramuscular injection of diazepam 10 mg and anisodamine 10 mg. Then, 10 min before stent removal, oral and laryngeal tetracaine spray anesthesia was administered.

Patients lay on a digital subtraction angiography (DSA) table and were administered oxygen; ECG monitoring was performed, and the head was biased to the right. Under fluoroscopy, a 0.035-inch hydrophilic membrane guidewire and 5F vertebral artery catheter were inserted through the mouth to reach the trachea. The guidewire was withdrawn and 5 ml of 2% lidocaine and 1 mg of adrenaline were quickly injected through the catheter. The catheter and guide wire enter one side of the main bronchus through the inner cavity of the stent. Then we exchanged the Amplatz guide wire, withdrew the catheter, entered the stent along the guide wire, took the hook and its long sheath to the upper or lower end of the stent, and extended the hook out of the sheath head for 2–3 cm. The stent removal method was determined according to whether the stent was covered, implantation time, granulation tissue proliferation, and patients' respiratory condition. The guidewire was always kept in the airway during stent removal. In cases of extensive bleeding, the catheter was quickly inserted along the guidewire, and 1 mg of adrenaline diluted with 9 ml of normal saline was locally sprayed (3–5 ml/time) through the catheter at the original stent implantation site to encourage the patient to cough and expectorate. If there was extensive bleeding, adrenaline was sprayed locally to stop bleeding. The guidewire could also be introduced into the sputum suction tube to extract the sputum in the suction duct and withdrawn when the patient had no hemoptysis or dyspnea and showed stable vital signs. After returning to the ward, the patients received hemostasis, expectorant, and anti-inflammatory treatments, and their vital signs, hemoptysis volume, and condition were closely observed.

### Statistical analysis

GraphPad Prism 5.0 was used for data processing and analysis. Quantitative data are expressed as mean ± standard deviation values. The results were expressed as mean ± standard deviation values.

## Results

One hundred and twelve patients (age: 7–85 [53.3 ± 16.7] years) were included in this study, including 61 male and 51 female patients. Overall, 148 tracheal stents were removed from 112 patients, including 103 and 45 covered and uncovered stents, respectively. Five patients suffered displacement after placement of the covered stent twice, then the tubular tracheal stent was removed and replaced in the same location. Seven patients with restenosis after stent removal underwent stent removal twice. Six patients underwent stent removal by the stent-in-stent technique. In four patients, stent displacement occurred after covered stent placement; these stents were replaced in the same location thrice. In two patients, stent displacement occurred after covered stent placement four times; these stents were replaced in the same location. In one case, the stent was replaced when displacement occurred after covered stent placement five times. The stent retention time was 70.3 ± 67.8 (range: 0–345) days (Table 1).

**Table 1:** Patient characteristics

Categories	Number of cases
Patients, no	112
Sex, Male	61 (54.5%)
Mean age, years	53.3 ± 16.7
*Disease*	
Airway fistula	22 (19.6%)
Airway stenosis	90(80.4%)
*Type of airway fistula*	
Esophageal airway	18 (81.8%)
Thoracogastric airway	3 (13.6%)
Tracheo-mediastinal	1 (4.5%)
*Type of airway stricture*	
Benign	63(70.0%)
Malignant	27(30.0%)
*Type of Benign airway stricture*	
Endotracheal intubation	20(74.1%)
Tracheotomy	4(14.8%)
Recurrent chondritis	1(3.7%)
Bronchial tuberculosis	2(7.4%)
*Type of tubular stent*	
Covered	103(69.6%)
Bare	45 (30.4%)

### Indication for stent removal

The indications of tracheal stent removal are shown in Table 2. The common reasons for the removal of tracheal stents are planned removal, excessive granulation tissue proliferation, and stent displacement. The planned stent retention time was 65.0 ± 34.9 (10–150) days. The retention times for excessive proliferation and stent displacement were 116.5 ± 84.9 (17–300) and 7.8 ± 3.6 (1–18) days, respectively. The retention times for broken and intolerable stents were 176.0 ± 102.1 (35–345) and 3.7 ± 3.1 (1–7) days, respectively. If the stent placement position was inappropriate, the stent was immediately replaced (day 0).

**Table 2:** Indications for stent removal

Complication	Number of cases
Planned removal	46(31.1%)
Granuloma formation	49(33.1%)
Migration	39(26.4%)
Stent fracture	6(4.1%)
Stent intolerance	3(2.0%)
Inappropriate location	5(3.4%)
Total	148(100.0%)

### Stent removal status

A total of 148 stents were removed from 112 patients at our center, of which 142 (95.9%) stents were completely removed and six (4.1%) stents were removed with a small amount of metal residue. A total of 78 (69.6%) patients underwent removal of 97 (65.5%) tracheal stents by the internal stripping method, in which the distal end of the stent was attached to a stent-removal hook and the stent was inverted, and 32 (21.6%) patients underwent removal of 39 (26.4%) tracheal stents by the direct removal method, in which the upper end of the stent was attached to a stent-removal hook and the proximal end of the stent was collapsed. Among these cases, in six (5.4%) patients with severe hyperplasia of granulation tissue after tracheal stent implantation, 12 (8.1%) tracheal stents were successfully removed by the stent-in-stent technique [10]. The overall operation time for stent removal was 28.9 ± 20.1 (9–111) min, while the planned operation time for stent removal was 28.7 ± 19.1 (11–87) min. The operation time for stent removal due to excessive granulation tissue hyperplasia, stent displacement, stent fracture, stent intolerance, and poor stent placement was 38.7 ± 27.5 (17–111) min, 22.2 ± 10.8 (12–47) min, 55.5 ± 16.5 (38–83) min, 25.3 ± 15.3 (16–43) min, and 14.2 ± 5.9 (9–24) min, respectively (Table 3).

**Table 3:** Relationship between the time of and indication for stent removal

Indications	Number of cases	Mean duration (median, range), minutes
Planned removal	46(31.1%)	28.7 ± 19.1 (11–87)
Granuloma formation	49(33.1%)	38.7 ± 27.5 (17–111)
Migration	39(26.4%)	22.2 ± 10.8 (12–47)
Stent fracture	6(4.1%)	55.5 ± 16.5 (38–83)
Stent intolerance	3 (2.0%)	25.3 ± 15.3(16–43)
Inappropriate location	5 (3.4%)	14.2 ± 5.9 (9–24)
Total	148(100.0%)	28.9 ± 20.1 (9–111)

### Complications associated with stent removal

Minor bleeding and a slight decrease in oxygen saturation during stent removal were defined as mild complications. A small amount of stent residue, a significant decrease in oxygen saturation, and a small amount of hemoptysis were defined as moderate complications. Massive hemoptysis, tracheal rupture, mediastinal fistula, and death were defined as serious complications. Mild complications occurred in 16 (14.3%) cases that showed mild mucosal bleeding during tracheal stent removal, and no rebleeding was observed after local application of adrenaline. Moderate complications occurred in 13 (11.6%) cases, including a small amount of stent wire residue in six (5.4%) cases, a significant decrease in oxygen saturation in six (5.4%) cases, and a small amount of hemoptysis in one (0.9%) case. Six (5.4%) stents were removed with the stent-removal hook under fluoroscopy, and the residual wires of the stents were successfully removed under bronchoscopy. In six (5.4%) cases, oxygen saturation decreased significantly during stent removal, and the patients underwent interventional tracheal intubation and respiratory balloon-assisted breathing [11]. After the patient's oxygen saturation normalized, the stent was exchanged again, the sheath was taken out, and the stent was successfully taken out through the sheath into the stent-removal hook. One (0.9%) patient showed hemoptysis of approximately 90 mL after stent removal. To prevent asphyxia caused by bleeding, tracheal intubation was performed 2 cm above the carina, and hemostatic drugs were injected intravenously. The hemoptysis stopped and the tracheal intubation was removed after the blood oxygen saturation stabilized.

### Follow-up

All patients were followed up in the clinic or by telephone. The follow-up period was one month after tracheal stent removal. During the follow-up period, a total of 50 tracheal stents were placed in 28 patients after airway stent removal, of which 38 stents were placed in 16 patients after stent removal due to stent displacement, five tracheal stents were replaced after removal because of poor position, and seven tracheal stents were replaced because of severe granulation tissue hyperplasia after stent implantation and dyspnea after removal. Four patients with tracheal stenosis developed severe dyspnea 10 days after airway stent removal. After tracheal intubation, they were sent to the respiratory ICU. The remaining 80 patients experienced no notable discomfort after tracheal stent removal. During the follow-up period, there were no complications related to stent removal, such as massive hemorrhage, perforation of the airway wall, mediastinal abscess, and death.

## Discussion

Tracheal metal stents are categorized as covered, partially covered, and uncovered. Stent restenosis, stent displacement, and stent rupture after granulation tissue formation are possible complications after tracheal metal stent implantation [4,12]. Prolonged indwelling of tracheal stents may increase the risk of complications. Rampey et al. [13] reported that after placement of the stent for more than 4–6 weeks, the stent will show endothelialization, making its removal more difficult. Fracture of metal fatigue stents is also related to the long-term placement of metal stents in the body [14]. This was indirectly explained by the retention time of 176.0 ± 102.1 (35–345) days for broken stents in the center. Therefore, timely removal of tracheal stents can avoid or reduce the incidence of tracheal stent complications.

The success rate of removing tracheal stents under fluoroscopy is high, and the incidence of serious complications is low, but tracheal stent removal can also be associated with complications such as failure to remove, residual stent wire, rupture of the tracheal mucosa, bleeding, tracheal perforation, reblockage, pneumothorax, respiratory failure, and even death [15]. The existing techniques for tracheal stent removal involve fluoroscopy and bronchoscopy. Song first reported the removal of tracheal stents under fluoroscopy, and subsequently reported that metal stents were removed under hard bronchus and a fiberoptic bronchoscope [16–18]. Alazem et al. [19] reported the experience of removing expanded metal tracheal stents under a rigid bronchoscope. This technique requires general anesthesia, and when the tracheal wall is too close to the stent, stent removal with a rigid bronchoscope is associated with the risk of mucosal bleeding. In contrast, removal of metal tracheal stents under fluoroscopic guidance does not require general anesthesia, and the damage to tracheal tissue is less than that under traditional endoscopy [4]. Over the past 15 years, 148 tracheal stents have been removed under fluoroscopy, and the success rate of complete removal of stents is 95.9% (129/135), which is much higher than the 27% success rate for complete removal of stents reported by Lunn et al. Moreover, this technique is associated with fewer complications, and it does not require destruction of the whole stent, resulting in fewer stent residues [7,19]. The costs of removing the tubular airway metal stent under fluoroscopy and bronchoscopy were as follows. The cost of the direct extraction method was about 4000 yuan. The cost of the internal stripping method was about 4000 yuan. The cost of the stent-in-stent method was about 9500 yuan. The cost of the bronchoscopic removal stent method was about 4500 yuan.

The time required for tracheal stent placement is closely related to the difficulty of removal and the occurrence of complications. A shorter placement time will result in less granulation tissue proliferation around the stent, easier stent removal, and fewer complications. Because stent removal should take into account the treatment effect, the duration of tracheal stent placement has been a topic of debate. Some studies have reported that stents in patients with tracheal stenosis are not easy to remove after more than 10 months, and that removal of stents within three months avoids complications in stent removal [17]. At our center, among the patients who underwent stent removal, the retention time for broken stent removal was 176.0 ± 102.1 (35–345) days, the removal time was 55.5 ± 16.5 (38–83) min, while the retention time in cases of poor stent placement was 0 days, and the removal operation time was 14.2 ± 5.9 (9–24) min. Thus, the longer the tracheal stent implantation time, the greater the possibility of stent fractures, and the longer the stent removal time, the greater the risk of serious complications.

Some precautions are essential for removing tracheal stents in this technique. First, before the stent is taken out, bronchoscopy must be performed to evaluate the proliferation of granulation tissue in and at both ends of the stent, and thereby determine the specific removal method. Patients showing severe granulation tissue hyperplasia in the stent were treated with microwave cauterization under a fiberoptic bronchoscope. Second, anti-inflammatory treatment and atomization inhalation are administered 1–3 days before the operation to reduce tracheal inflammation and promote sputum discharge, and thereby facilitate the removal of the tracheal stent. Third, skilled and adequate hemostatic treatment is key to the removal of tracheal stents. When hanging the stent with the stent-removal hook, the length of the removal hook extending out of the sheath should be carefully managed to avoid penetrating the tracheal wall and causing a mediastinal abscess. Hemostatic drugs should be sprayed before hanging the stent; the catheter should be quickly exchanged after taking out the stent; and hemostatic drugs should be sprayed at the original stent through the catheter, which can prevent local tissue bleeding. Fourth, in cases showing severe dyspnea or massive bleeding during the stenting process, interventional technology can be used to quickly intubate the trachea and administer symptomatic treatment to avoid asphyxia.

In conclusion, fluoroscopic interventional techniques are feasible, safe, and effective methods to remove tracheal stents, making them worthy of clinical application. Nevertheless, this was a single-center study with no control group. Therefore, future studies should aim to further explore optimal stent removal time and measures to avoid complications.

## Data Availability

The datasets used and analyzed during this study are available from the corresponding author on reasonable request.
